# Effects of robotic-assisted early mobilization versus conventional mobilization in intensive care unit patients: prospective interventional cohort study with retrospective control group analysis

**DOI:** 10.1186/s13054-024-04896-1

**Published:** 2024-04-06

**Authors:** Lucas Huebner, Angelika Warmbein, Christina Scharf, Ines Schroeder, Kirsi Manz, Ivanka Rathgeber, Marcus Gutmann, Johanna Biebl, Amrei Mehler-Klamt, Jana Huber, Inge Eberl, Eduard Kraft, Uli Fischer, Michael Zoller

**Affiliations:** 1 Department of Anaesthesiology, LMU University Hospital, LMU Munich, Munich, Germany; 2 Clinical Nursing Research and Quality Management Unit, LMU University Hospital, LMU Munich, Munich, Germany; 3 Institute for Medical Information Processing, Biometry and Epidemiology (IBE), Faculty of Medicine, LMU Munich, Munich, Germany; 4 Department of Orthopaedics and Trauma Surgery, Musculoskeletal University Center Munich (MUM), LMU University Hospital, LMU Munich, Munich, Germany; 5 Professorship for Nursing Science, Faculty of Social Work, Catholic University of Eichstätt-Ingolstadt, Eichstätt, Germany

**Keywords:** Early mobilization, Intensive care, Critical care, ICU-acquired weakness, Robotics

## Abstract

**Background:**

Approximately one in three survivors of critical illness suffers from intensive-care-unit-acquired weakness, which increases mortality and impairs quality of life. By counteracting immobilization, a known risk factor, active mobilization may mitigate its negative effects on patients. In this single-center trial, the effect of robotic-assisted early mobilization in the intensive care unit (ICU) on patients’ outcomes was investigated.

**Methods:**

We enrolled 16 adults scheduled for lung transplantation to receive 20 min of robotic-assisted mobilization and verticalization twice daily during their first week in the ICU (intervention group: IG). A control group (CG) of 13 conventionally mobilized patients after lung transplantation was recruited retrospectively. Outcome measures included the duration of mechanical ventilation, length of ICU stay, muscle parameters evaluated by ultrasound, and quality of life after three months.

**Results:**

During the first week in the ICU, the intervention group received a median of 6 (interquartile range 3–8) robotic-assisted sessions of early mobilization and verticalization. There were no statistically significant differences in the duration of mechanical ventilation (IG: median 126 vs. CG: 78 h), length of ICU stay, muscle parameters evaluated by ultrasound, and quality of life after three months between the IG and CG.

**Conclusion:**

In this study, robotic-assisted mobilization was successfully implemented in the ICU setting. No significant differences in patients’ outcomes were observed between conventional and robotic-assisted mobilization. However, randomized and larger studies are necessary to validate the adequacy of robotic mobilization in other cohorts.

*Trial registration*: This single-center interventional trial was registered in clinicaltrials.gov as NCT05071248 on 27/08/2021.

**Supplementary Information:**

The online version contains supplementary material available at 10.1186/s13054-024-04896-1.

## Background

Approximately every third survivor of critical illness suffers from intensive-care-unit-acquired weakness (ICUAW) [1]. Affected patients show a reduction in muscle mass, strength, and function and suffer from increased mortality and impaired quality of life [2]. Known risk factors are mechanical ventilation, severe sepsis, organ dysfunctions, neuro/myotoxic agents, and immobilization [3]. By specifically counteracting immobilization, active mobilization may mitigate some negative effects of ICUAW [4–8].

The effects of robotic-assisted mobilization on the critically ill have not yet been investigated. In the rehabilitation after stroke, addition of robotic-mobilization increases the chances of regaining independent walking abilities especially if applied early and in the non-ambulatory [9]. If robotic assistance can reasonably be integrated into the intensive care setting, it could support early mobilization and relieve mobilizing professionals. The aim of this study was to explore the effects of robotic mobilization and verticalization on the outcomes of critical care patients and compare them to a conventionally mobilized historical cohort. The primary outcome measure was the duration of mechanical ventilation. Secondary outcome measures were the length of ICU stay, muscle parameters, and quality of life after three months.

## Methods

This project was part of the MobiStaR project (Mobilization of intensive care patients by a new standard in adaptive robotics, NCT05071248), which evaluated robotic-assisted mobilization in the surgical intensive care unit (ICU) and the reception by critical care providers [10].

Eligible patients were adults (> 18 years) scheduled for elective surgery with an expected ventilation duration of > 48 h. We excluded patients not capable of consent, chronically bedridden patients, patients with preoperative ventilation, and patients with preexisting neuromuscular disease that impaired strength. A control group was retrospectively selected from adults that had undergone elective surgery between May and August 2021, met the same inclusion and exclusion criteria as the intervention group, and had received ultrasound measurements routinely or as part of another observational study [11]. No matching was performed. A full list of inclusion and exclusion criteria was published with the study protocol [10].

Twice daily, the treating ICU team was consulted, whether robotic mobilization was deemed feasible. If deemed feasible, patients received 20 min of robotic-assisted verticalization and mobilization twice daily for up to 10 sessions or seven days utilizing the VEMOTION® robot (Reactive Robotics, Munich), which allows continuous verticalization up to 70° and cyclic stepping movements. Verticalization was increased according to patient comfort; unconscious patients were verticalized up to about 30°. If robotic mobilization was not deemed feasible by the primary care team, conventional mobilization was performed. The retrospectively recruited CG was mobilized per usual care, i.e. up to two sessions per day, adapted to the patient's condition and performed by a team of physiotherapists and nurses according to standardized mobilization guidelines.

Musculus quadriceps femoris (MQF) thickness, musculus rectus femoris (MRF) cross-sectional area (CSA), diaphragm thickness and mobility were measured via ultrasound as previously described [12–14]. Measurements were taken preoperatively, on days one to three, after one week when still in the ICU, and after three months. Quality of life was assessed three months after ICU admission using the German Short Form-36 (SF-36) questionnaire [15]. Functional Status Score for the Intensive Care Unit (FSS-ICU) [16] was determined by the treating physiotherapist or nurse. Cumulative doses of analgetic medication, laxatives, and insulin were extracted from the patient data management system.

Continuous variables were summarized by median (interquartile range), categorical variables by frequency [percentage]; Continuous variables were compared between groups using the Mann–Whitney *U* test and categorical variables were compared using Fisher’s exact test. Time-to-event variables were plotted as Kaplan–Meier curves and compared using the log-rank test. Linear mixed effects models with the patient as a random effect were estimated for the muscle parameters. Medication use and cumulative doses during ICU stay were collected from the electronic patient data system. All statistical analyses were performed using R (Version 4.2.2).

## Results

From September 2021 to March 2022, 23 patients scheduled for lung transplantation surgery were enrolled in the intervention group (IG). One patient withdrew consent for all data and was excluded. Additional six patients were excluded from the analysis because robotic-assisted mobilization was not possible during the first seven days because they were deemed too critically ill by the treating ICU team (see Additional file 1: fig. S1). Median SAPS II in this group was 46. Of 18 admissions meeting the inclusion criteria, 13 had received ultrasound measurements and were retrospectively included in the control group (CG). There were no statistically significant differences in patient characteristics between IG and CG at baseline (see Table 1).

**Table 1:** Patient characteristics

	Intervention (*n* = 16) *n* [%] or median (IQR)	Control (*n* = 13) *n* [%] or median (IQR)	P*
Male sex	8 [50%]	10 [77%]	0.249
Age in years	61 (55–63)	58 (49–63)	0.538
Height in cm	169 (165–173)	174 (168–178)	0.163
Weight in kg	64 (58–71)	69 (59–77)	0.908
Body mass index in kg/m^2^	23 (18–30)	23 (19–26)	0.440
Pre-op. FSS-ICU	35 (35–35)	35 (34–35)	0.999
Diagnosis subgroup			
Chronic obstructive pulmonary disease	2	3	0.632
Interstitial lung disease, idiopathic pulmonary fibrosis	13	8	0.406
Cystic fibrosis	1	1	0.999
Primary pulmonary hypertension	0	1	0.448
Co-morbidities			
Arterial hypertension	6	3	0.454
Coronary heart disease	3	3	0.999
Diabetes mellitus	1	2	0.573
Chronic kidney disease	1	1	0.999
SAPS II	37 (32–42)	32 (24–37)	0.056
RASS (Day 1)	− 5 (− 5 to − 5)	− 5 (− 5 to − 5)	0.217
SOFA (Day 1)	7 (6–8)	8 (7–8)	0.671

Data are presented as a number [percent] or the median (interquartile range (IQR))

^*^Intervention group versus control group

### Mobilization

The median time from admission to first mobilization was 18 h in the IG and 13 h in the CG. No significant difference was detected. The median time to first robotic mobilization was 26 h. Patients mobilized with robotic assistance received a median of 6 (IQR: 3–8) mobilizations.

### Primary outcome

The median duration of ventilation was 126 h in the intervention group and 78 h in the control group (see Table 2). No significant difference was found (see Additional file 1: fig. S2).

**Table 2:** Patient outcomes

	Intervention (*n* = 16) median (IQR)	Control (*n* = 13) median (IQR)	P*
Length of invasive ventilation in h	126 (85–330)	78 (57–151)	0.181
Length of ICU stay in days	14 (8–21)	8 (7–10)	0.187
No. robotic-assisted mobilizations	6 (3–8)	–	NA
FSS-ICU at discharge	32 (29–35)	34 (30–34)	0.671

Data are presented as median (interquartile range (IQR))

*Intervention group versus control group

### Secondary outcomes

No significant difference in ICU length of stay was observed between patients receiving robotic-assisted mobilization (median 14 days (IQR 8–21)) and controls (median 8 days (IQR 7–10), *p* = 0.187). In patients receiving low or no pressure support, a trend toward increased diaphragm mobility was observed from day two until three months postoperatively in both groups (see Additional file 1: fig. S3). Low pressure support was defined as positive end-expiratory pressure ≤ 5cmH_2_O and pressure support ≤ 5cmH_2_O. In a linear mixed univariate model, diaphragm motility was positively associated with a higher functional status score (FSS-ICU) (Estimator: 0.02; 95% CI (0.00, 0.04); *P* = 0.0345) and negatively associated with a higher SOFA score (Estimator: − 0.10; 95% CI (− 0.16, − 0.03); *P* = 0.006). No significant differences in diaphragm thickness, musculus quadriceps femoris (MQF) thickness or musculus rectus femoris (MRF) CSA were observed between the groups (see Additional file 1: fig. S4). Compared to preoperative measurements of MQF thickness, a median decrease of 11% and 9% was seen on day seven in the CG and IG, respectively. However, at three months, the CG showed a median increase of 13%, while the IG stayed decreased by 5%.

On day one, a median increase of 8% in MRF CSA in the CG and 10% in the IG was seen. By day seven, the CG returned to preoperative measurements, while the IG showed almost no decline. At three months, an overall increase of 13% and 10% was observed in the IG and CG, respectively.

There were no statistically significant differences between the two groups in the eight individual dimensions of health or in the physical and psychological health component sum score measured with the SF-36 after three months (see Additional file 1: fig. S5).

Cumulative doses of analgetic medication, laxatives, and insulin in the IG were comparable with those in the controls (see Additional file 1: Table S1). There was no significant difference in the doses of the sedatives propofol and midazolam, yet dexmedetomidine was used significantly more often in the IG (median 2.7 mg versus 0.0 mg; *p* = 0.006).

## Discussion

This study is the first to report outcomes after robotic-assisted mobilization in the ICU. With the inclusion criteria described, only lung transplant recipients, a small subset of ICU patients with a high degree of homogeneity, were enrolled. Six patients did not receive robotic-assisted mobilization, as they were not considered stable enough by the treatment team, and one patient withdrew consent. This illustrates that robotic mobilization is not appropriate for every patient and that patient-tailored, high-quality conventional mobilization remains essential. Nevertheless, robotic-assisted mobilization was possible in the majority of patients within the first seven days.

No significant differences in ICU length of stay or time on invasive ventilation were seen in patients who received robotic-assisted mobilization. The most recent gold standard, the TEAM RCT showed no difference in ICU length of stay between increased early mobilization and standard care [17]. Diaphragm dysfunction was correlated with prolonged weaning and longer ICU stay [18]. In this study, diaphragm motility increased during ICU stay and beyond regardless of group and was correlated with FSS-ICU and inversely correlated with severity of illness.

We observed a reduction in median MQF thickness of approximately 10% during the first 7 days in both groups, which is in line with previous reports of 16% decline within the first week of ICU stay [13]. Puthucheary et al. reported a 12.5% decrease in MRF CSA from day one to day seven [19], which is similar to what we observed in our control group. Physical functioning is significantly reduced in patients with ICUAW [2] and has been shown to be improved by rehabilitation therapy [20]. In this study, no significant differences in quality of life were observed at 3 months, which is consistent with studies showing no differences in quality of life between standard and early or intensive mobilization [5, 21].

The limitations of the study are: First, this was a single-center design and included only lung transplant recipients, a unique but homogeneous group. Second, enrollment during the COVID pandemic limited the number of patients available, introducing the possibility of not detecting true differences due to small sample size. Third, the control population was recruited retrospectively, which introduces the risk of selection bias and was limited by the need for documented ultrasound measurements.

In conclusion, robotic-assisted early mobilization of critical care patients showed no significant differences in patients’ outcomes when compared to conventional mobilization. Thus, no apparent negative effects of robotic-assisted mobilization on patients’ outcome were found. Further interventional studies with larger groups are needed to compare the outcomes of robotic and conventional mobilization in other cohorts.

### Supplementary Information

**Additional file 1**. **Supplemental figure 1**: Flowchart of inclusion and exclusion of patients.

## Data Availability

The datasets used in this study are not publicly available due to IRB agreements but are available from the corresponding author upon reasonable request.

## Abbreviations

CG: Control group
CSA: Cross-sectional area
FSS-ICU: Functional Status Score for the Intensive Care Unit
ICU: Intensive care unit
ICUAW: Intensive-care-unit-acquired weakness
IG: Intervention group
MobiStaR: Mobilization of intensive care patients by a new standard in adaptive robotics
MQF: Musculus quadriceps femoris
MRF: Musculus rectus femoris
SAPS: Simplified Acute Physiology Score
SF-36: Short Form-36 questionnaire
SOFA: Sequential Organ Failure Assessment
