# Supplementary material for: Effects of robotic-assisted early mobilization versus conventional mobilization in intensive care unit patients: prospective interventional cohort study with retrospective control group analysis
Source: Crit Care. 2024 Apr 6;28:112. doi: 10.1186/s13054-024-04896-1 (PMC10999075; doi:10.1186/s13054-024-04896-1)
Supplement: Supplementary file 1 — Additional file 1. Supplemental figure 1: Flowchart of inclusion and exclusion of patients. [file 13054_2024_4896_MOESM1_ESM.docx]

Supplements:

18 admissions meeting criteria

5 excluded for missing ultrasound measurements

13 Historical controls

23 patients enrolled for intervention

7 were excluded

1 withdrew consent for all data

6 received no robotic mobilization, as too ill for robotic mobilization

16 patients with robotic mobilization

Supplemental figure 1: Flowchart of in and exclusion of patients


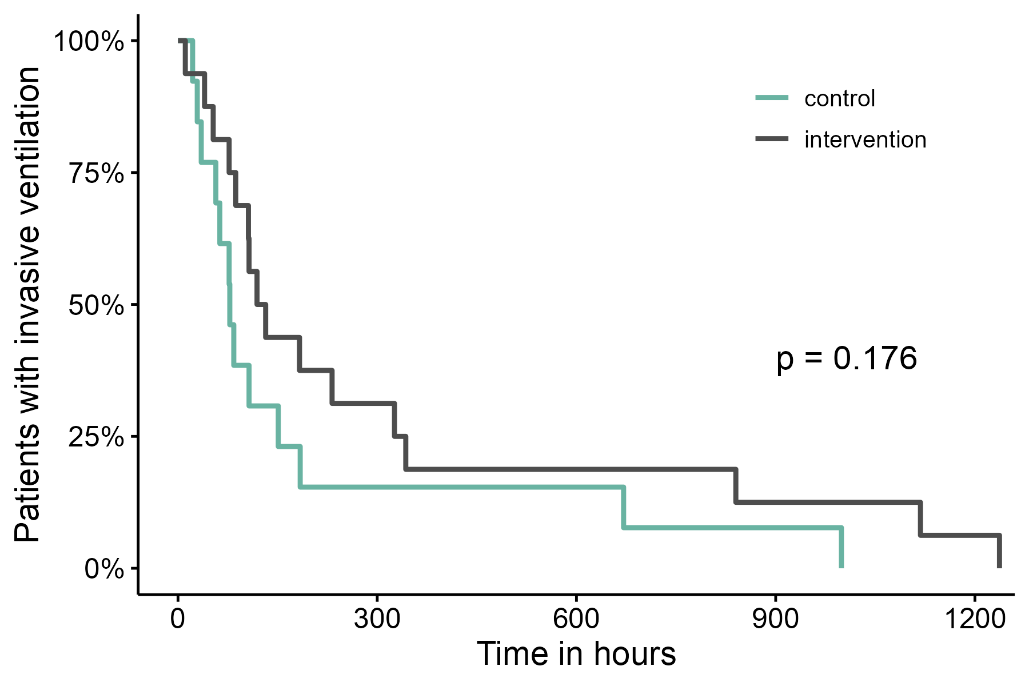


Supplemental figure 2: Kaplan–Meier curves displaying the duration of invasive ventilation in the intervention and control groups.


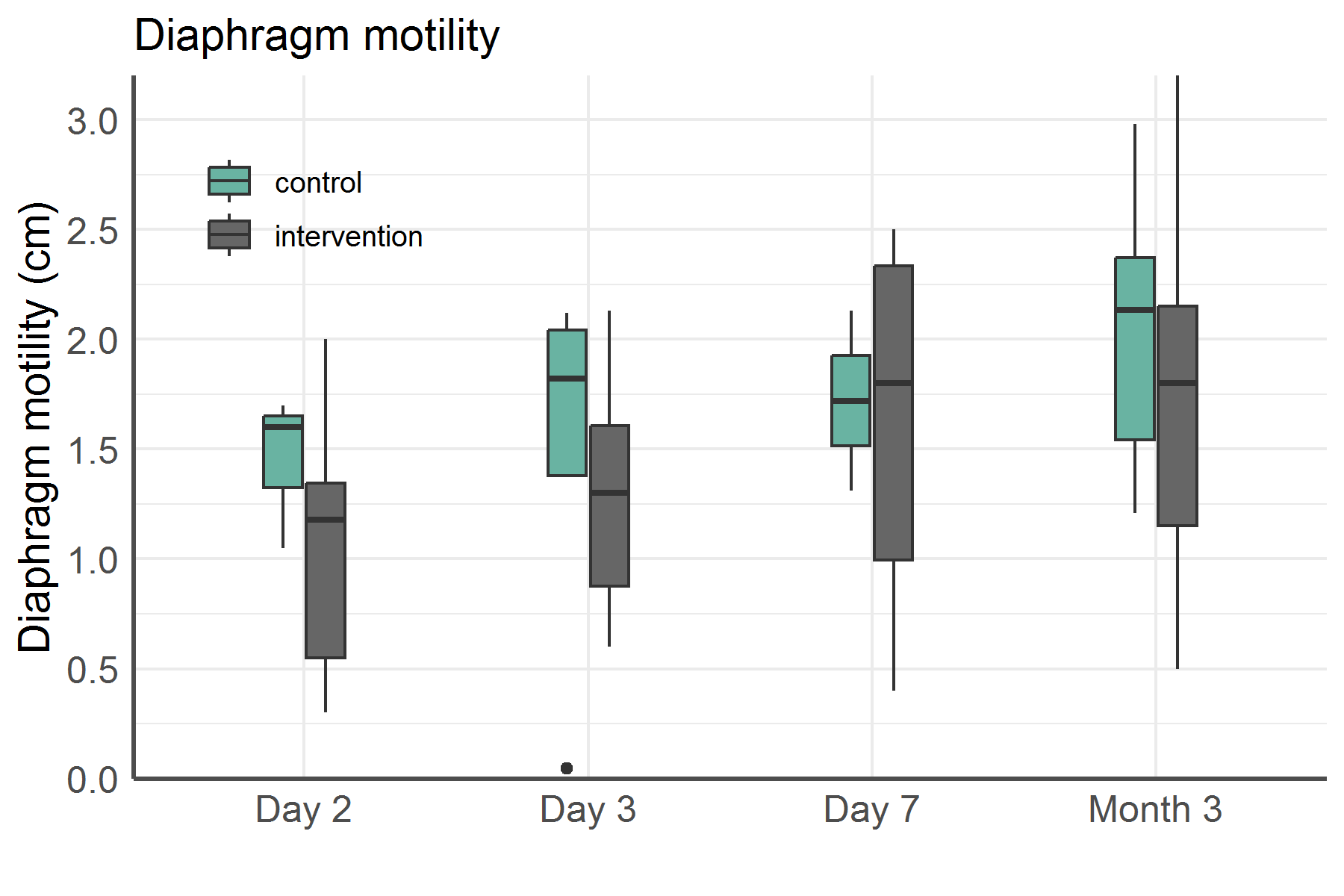


Supplemental figure 3: Diaphragm motility in non-invasively ventilated patients

Diaphragm motility of conventionally and robotically assisted mobilized, non-invasively ventilated patients over time.


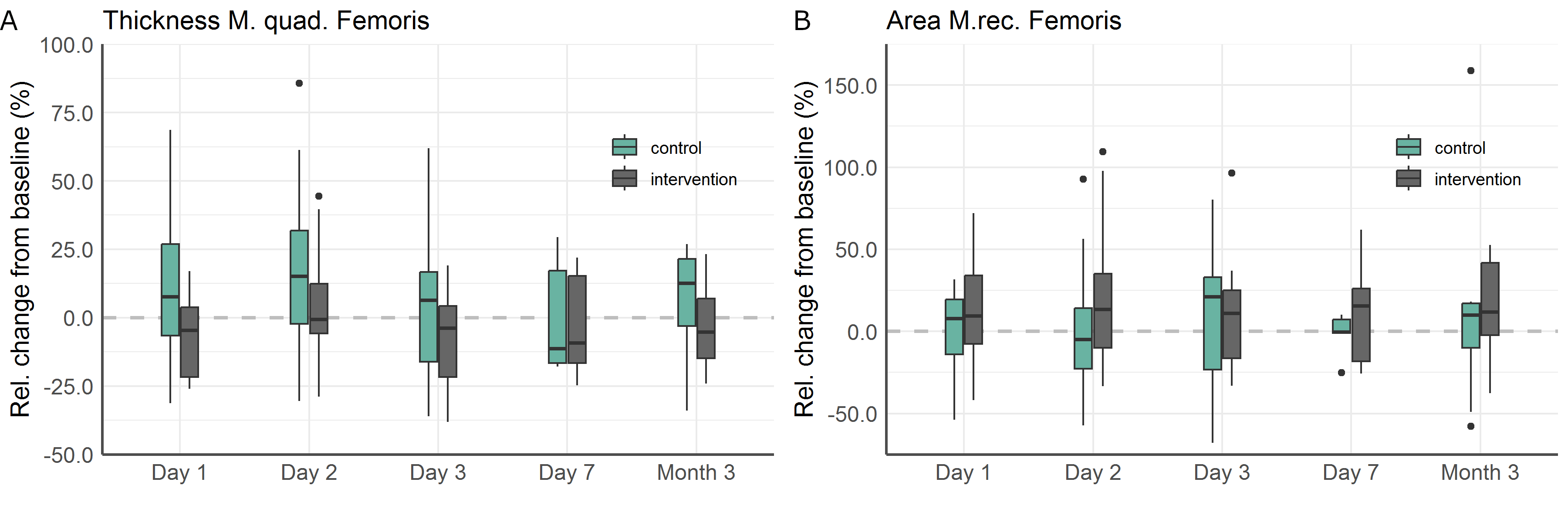


Supplemental figure 4: Relative change in the thickness of M. quadriceps femoris (A) and cross-sectional area of M. rectus femoris (B). (Normalized to preoperative Measurements)


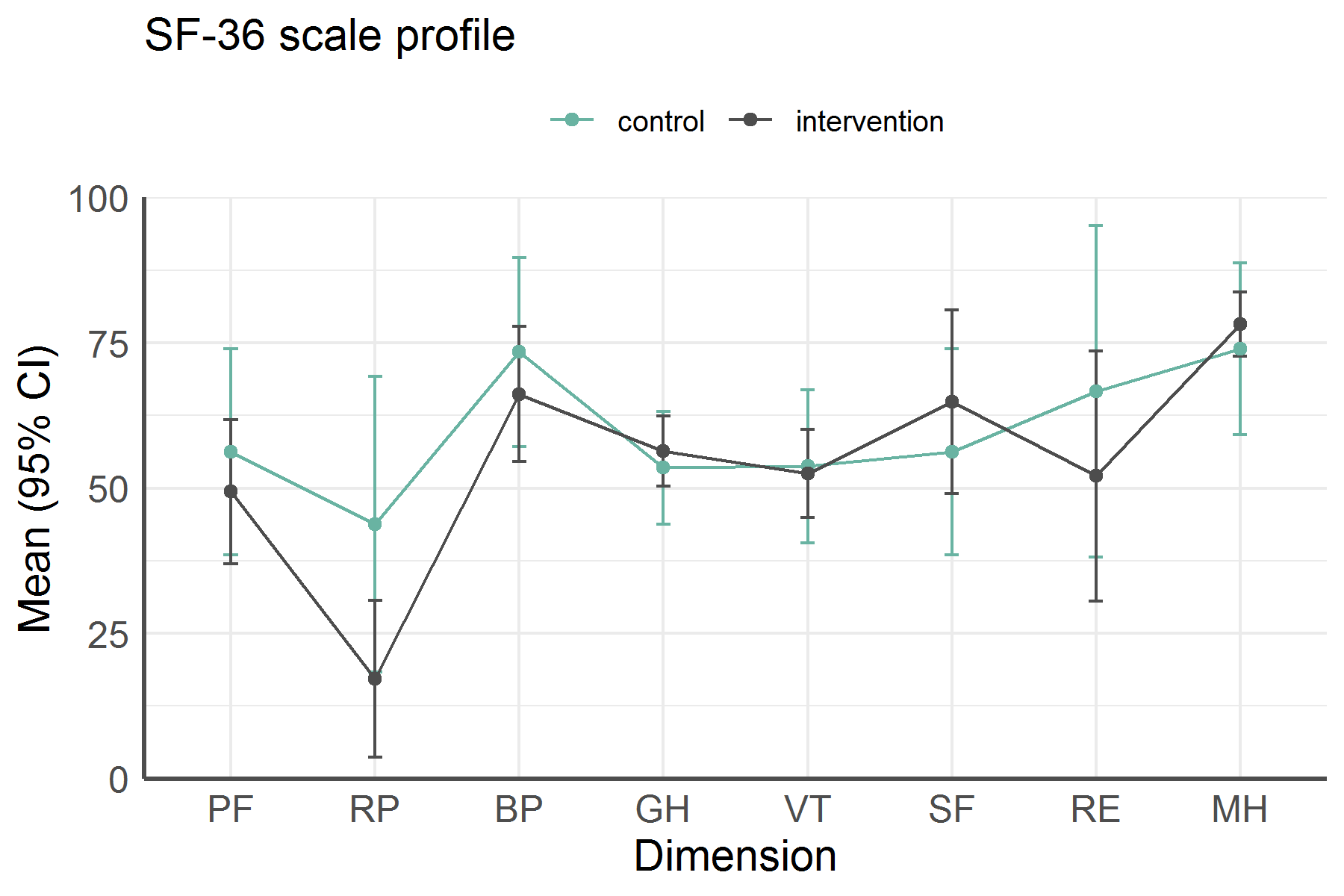
Supplemental figure 5: Quality of life: Scale profile of the SF-36 questionnaire after three months

Means are presented as dots, standard deviations as vertical bars. Abbreviations: PF: Physical Functioning, RP: Role: Physical, BP: Bodily Pain, GH: General Health, VT: Vitality, SF: Social Functioning, RE: Role: Emotional, MH: Mental Health.

Supplemental table 1: Cumulative drug consumption during days 0 to 7 per drug and group

|  | All  (n=29) | Intervention  (n=16) | Control  (n=13) | p |
| --- | --- | --- | --- | --- |
| Sufentanil (µg) | 1045  (607 – 2508) | 1063  (643 – 2409) | 1045  (588 – 2508) | 0.983 |
| Piritramide (mg) | 15  (0 – 38) | 14  (0 – 38) | 15  (9 – 41) | 0.722 |
| Propofol (mg) | 8524  (3814 – 17489) | 7181  (4008 – 18477) | 9508  (2736 – 14984) | 0.913 |
| Midazolam (mg) | 0  (0 – 0) | 0  (0 - 0) | 0  (0 – 235) | 0.183 |
| Dexmedetomidine (mg) | 0.2  (0.0 – 3.3) | 2.7  (0.0 – 4.3) | 0.0  (0.0 – 0.2) | **0.006** |
| Naloxone (mg) | 160  (96 – 216) | 184  (112 – 224) | 152  (96 – 208) | 0.282 |
| Movicol (bags) | 6  (4 – 10) | 7  (5 – 9) | 6  (4 – 11) | 0.642 |
| Sodium picosulfate (mg) | 23  (15 – 45) | 30  (15 – 47) | 23  (8 – 45) | 0.354 |
| Neostigmine (mg) | 0.9  (0 – 1.5) | 0.9  (0.0 – 1.5) | 0  (0 – 1.5) | 0.798 |
| Insulin (IE) | 85  (27 – 126) | 89  (21 – 123) | 44  (28 – 180) | 0.913 |

Median (minimum - maximum). The p-value refers to the comparison of the intervention group vs. control group. Statistically significant p-values are printed in bold.
